# Bone Marrow Infection by *Pneumocystis jirovecii* in a Patient with AIDS: A Case Report and Literature Review

**DOI:** 10.3390/idr17030047

**Published:** 2025-05-02

**Authors:** Diego Alejandro Cubides-Diaz, Valentina Negrette-Lazaro, Viviana Poveda-Hurtado, Juan Pablo López-Salazar, Carlos Mauricio Calderón-Vargas, Carlos Arturo Álvarez-Moreno

**Affiliations:** 1 Department of Internal Medicine, Universidad Nacional de Colombia, Bogotá 111321, Colombia; 2 Faculty of Medicine, Universidad del Rosario, Bogotá 111221, Colombia; valentina.negrette@urosario.edu.co; 3 Department of Internal Medicine, Universidad de La Sabana, Chía 250001, Colombia; vivianapohu@unisabana.edu.co; 4 Department of Internal Medicine, Hospital Universitario de La Samaritana, Zipaquirá 250252, Colombia; juanplopez9504@gmail.com; 5 Department of Internal Medicine, Hospital Universitario de La Samaritana, Bogotá 110411, Colombia; caldecal@gmail.com; 6 Faculty of Medicine, Universidad Nacional de Colombia, Bogotá 111321, Colombia; calvarez@colsanitas.com; 7 Clínica Universitaria Colombia, Clínica Colsanitas S.A., Bogotá 111321, Colombia

**Keywords:** *Pneumocystis jirovecii*, extrapulmonary infection, bone marrow, AIDS, opportunistic infections, case report, pancytopenia

## Abstract

Background: *Pneumocystis jirovecii* primarily causes pneumonia in immunosuppressed individuals, particularly those living with advanced HIV/AIDS. Extrapulmonary dissemination is uncommon, with bone marrow involvement described in only a handful of cases globally. Bone marrow infection occurs in the setting of severe immunosuppression, poses diagnostic challenges, and carries a high mortality rate. Methods: We describe the case of a 34-year-old man newly diagnosed with HIV/AIDS, presenting with severe immunosuppression and *Pneumocystis jirovecii* pneumonia. The patient initially improved with cotrimoxazole and corticosteroids, but was readmitted shortly after discharge with abdominal pain, diarrhea, and worsening pancytopenia. A bone marrow biopsy revealed *Pneumocystis jirovecii* cysts, confirming disseminated infection. Concomitant Kaposi sarcoma involving the skin and gastrointestinal tract was also diagnosed. Despite antimicrobial therapy, the patient’s condition worsened, leading to multisystem organ failure and death two months later. Conclusions: This case highlights a rare presentation of disseminated *Pneumocystis jirovecii* infection with bone marrow involvement in a patient with advanced HIV/AIDS. Although infrequent, this complication should be considered in individuals with *Pneumocystis jirovecii* pneumonia who develop persistent cytopenias and systemic symptoms. Diagnosis depends on histopathologic confirmation, which may lead to under-recognition. Early suspicion and individualized management are essential, though the optimal treatment approach for extrapulmonary infection remains undefined.

## 1. Introduction

*Pneumocystis jirovecii* (*P. jirovecii*) is an obligate fungal pathogen with parasitic behavior that colonizes the human respiratory tract and is primarily transmitted through airborne human-to-human contact. *P. jirovecii* can cause life-threatening pneumonia in individuals with impaired immunity such as those with advanced HIV/AIDS (particularly with CD4 counts <200 cells/μL), hematologic malignancies, solid organ transplants, or those receiving immunosuppressive therapies [1]. *P. jirovecii* pneumonia (PCP)—still commonly referred to by the acronym PCP to maintain consistency across the literature—remains a major opportunistic infection among people living with HIV, but its impact is increasingly recognized in non-HIV immunocompromised populations [1,2]. Genotyping studies have demonstrated that more than 90% of PCP cases involve genetically distinct mixtures of *P. jirovecii* strains, supporting the hypothesis that ongoing exposure and repeated inhalation from diverse sources—rather than the reactivation of latent organisms—is the primary mechanism driving PCP pathogenesis [1].

In recent years, PCP has emerged as a critical concern among patients with hematologic malignancies, solid tumors, organ transplants, and those receiving immunosuppressive therapies [2]. Unlike the subacute clinical course typically observed in HIV-positive individuals, PCP in non-HIV patients presents with a rapid onset and progression to respiratory failure, often within days. These patients frequently experience more severe hypoxemia, and a higher mortality rate compared to their HIV-infected counterparts. Diagnostic challenges are more pronounced in the non-HIV population due to a lower fungal burden. Radiologic findings also differ, ranging from classical diffuse ground-glass opacities to more sharply demarcated panlobular patterns [2]. Therapeutically, cotrimoxazole remains the first-line treatment; however, the optimal dosing and duration in non-HIV patients are not fully standardized. The benefit of adjunctive corticosteroids in this population also remains controversial [2]. Extrapulmonary infection by *P. jirovecii* is rare, with the lymph nodes and spleen being the most frequently affected sites [3]. Bone marrow involvement has been described only in a limited number of cases worldwide [3,4,5,6,7,8,9,10,11,12,13]. The primary risk factor associated with bone marrow infection is HIV/AIDS, particularly in patients who have not received adequate pharmacologic prophylaxis for *P. jirovecii*, such as systemic cotrimoxazole or dapsone. Inhaled pentamidine, although once widely used, has been associated with an increased risk of extrapulmonary disease due to insufficient systemic levels [3,4]. This stage of infection is believed to represent hematogenous or lymphatic dissemination from a primary pulmonary focus and carries a poor prognosis, with mortality rates reported to exceed 90% in several series [3,4,5,6,7,8,9,10,11,12]. In the comprehensive review by Ng et al., most cases of disseminated *P. jirovecii* with bone marrow involvement occurred in patients with primary pulmonary disease and were diagnosed postmortem [13]. Diagnosis typically relies on histopathologic examination, and in many instances, the condition is identified incidentally during the evaluation of tissue samples [3,4,5,6,7,8,9,10,11,12,13].

On the other hand, Kaposi sarcoma (KS) is a vascular tumor associated with human herpesvirus 8 (HHV-8) in patients living with HIV. It typically affects the skin, mucosa, and lymph nodes, but can also present with visceral dissemination, particularly involving the lungs and gastrointestinal tract, which worsens the prognosis [14]. KS may also be associated with hyperinflammatory syndromes such as immune reconstitution inflammatory syndrome (KS-IRIS) and inflammatory cytokine syndrome (KICS). KS-IRIS can present either by exacerbating pre-existing lesions or unmasking subclinical disease, often leading to rapid clinical deterioration. Unlike IRIS caused by other opportunistic infections, corticosteroids should be avoided in KS-IRIS due to the risk of accelerating viral replication and tumor progression [14]. KICS, a more recently recognized condition, is characterized by systemic inflammatory response syndrome (SIRS) with high levels of HHV-8 viremia and elevated interleukins (IL-6 and IL-10). Clinically, KICS often overlaps with multicentric Castleman disease (MCD), although without histological confirmation of MCD. KICS carries a high mortality rate and may occur concurrently with KS-IRIS, as both conditions are driven by immune dysregulation in the setting of viral replication. Prompt recognition and differentiation between these KS-driven complications are essential to guide appropriate management [14].

We present the case of an adult living with HIV who developed PCP, which subsequently progressed to disseminated disease with bone marrow involvement. During hospitalization, a concurrent diagnosis of KS affecting the skin and gastrointestinal tract was established, further complicating the clinical course. A summary of the patient’s clinical presentation and laboratory findings is provided below.

## 2. Case Report

**Figure 1:**
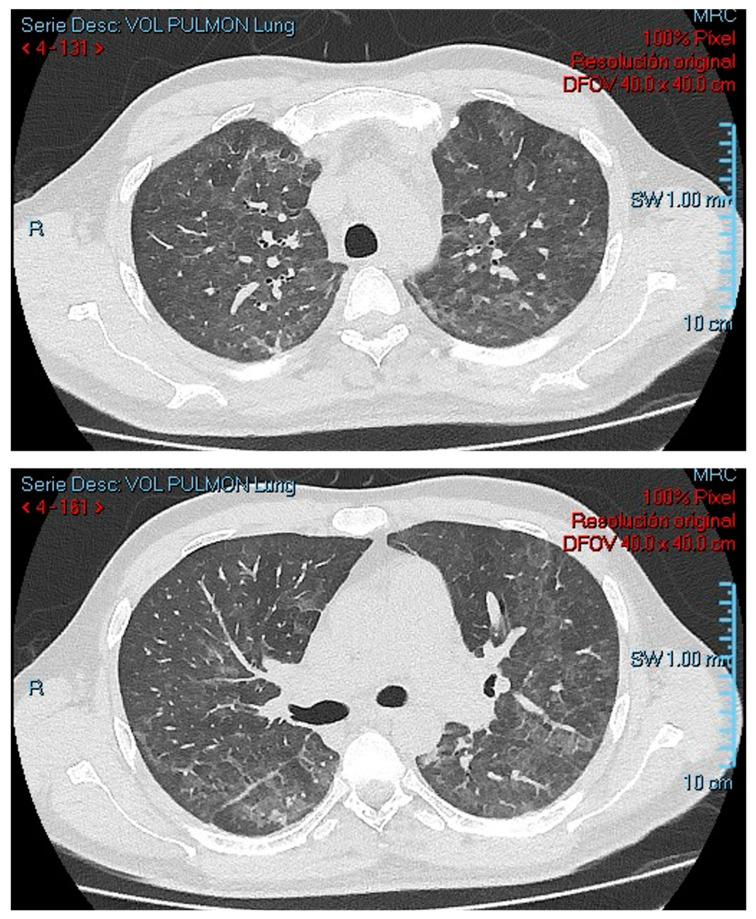
A computed tomography of the chest showing multiple ground glass opacities randomly distributed in both lung fields.

A 34-year-old man with a history of hypertension and unprotected sexual activity presented with a three-month history of fatigue, night sweats, an 11-pound involuntary weight loss, dry cough, intermittent non-dysenteric diarrhea, and progressive dyspnea. On examination, he was dehydrated, had an oxygen saturation of 78%, and exhibited oral lesions suggestive of candidiasis. Initial laboratories revealed mild thrombocytopenia, a positive four generation HIV test, reactive serologic tests for *Treponema pallidum*, and a blood arterial gas test with a moderate oxygenation disorder and a high alveolar–arterial gradient (Table 1). A chest X-ray showed left basal interstitial opacities, later characterized on chest tomography, revealing extensive ground-glass opacities randomly distributed in both lung fields (Figure 1).

**Figure 2:**
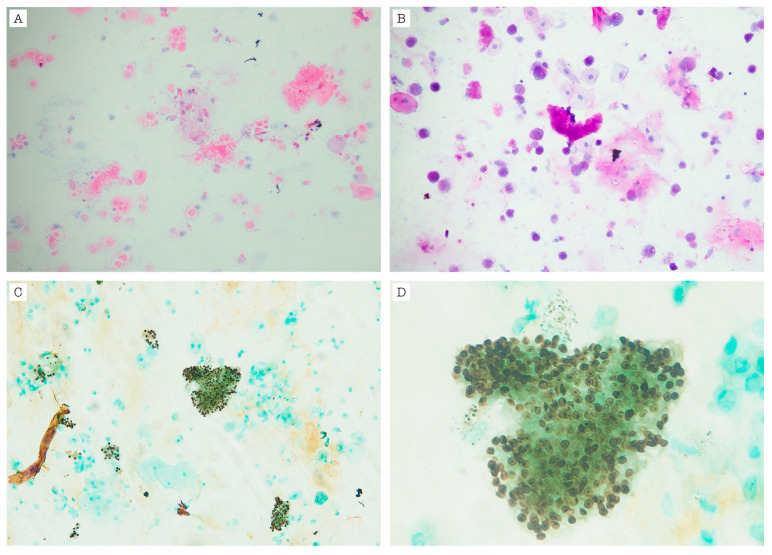
Bronchoalveolar lavage with, as follows: (**A**) Hematoxylin-eosin stain revealing foamy and cottony amorphic exudates; (**B**) PAS (Periodic Acid Schiff) stain highlights round structures with a small central condensation suggestive of *P. jirovecii* cysts; (**C**,**D**) GMS (Grocott–Gömöri’s methenamine silver) stain with magnification revealing dark brown round structures with a “folded spheres” or “flattened beach balls” appearance suggestive of *P. jirovecii* cysts.

The patient was considered to have a diagnosis of HIV infection, latent syphilis, oral candidiasis, and multilobar pneumonia of an undetermined etiology. The CD4 count was 33 cells/mm^3^, and the HIV viral load was 2,095,776 copies/mL. Due to the immunosuppressive condition and the tomographic pattern, a PCP was suspected. Antimicrobial therapy was started with fluconazole, benzathine penicillin, and cotrimoxazole with corticosteroids. The fibrobronchoscopy and bronchoalveolar lavage revealed structures suggestive of *P. jirovecii* (Figure 2), confirming the diagnosis of severe PCP and ruling out other infectious conditions. Therapy with cotrimoxazole was continued at a dose of 20 mg/kg/day, based on the trimethoprim component, as well as prednisolone on a tapering regimen. After 12 days of hospitalization, the patient was discharged on oral cotrimoxazole and prednisolone for 21 days, with plans to initiate antiretroviral therapy as an outpatient. The platelet count continued to be low, and other opportunistic infections, such as tuberculosis and histoplasmosis, were ruled out (Table 2).

Five days after discharge, the patient was readmitted for persistent abdominal pain, diarrhea, emesis, and fever. On readmission, laboratory tests showed persistent thrombocytopenia, leukocytosis, neutrophilia, elevated C-reactive protein, and hyponatremia (Table 1). Stool samples were negative for parasitic, bacterial, or fungal infection, and abdominal computed tomography revealed splenomegaly and an inflammatory thickening of the distal ileum.

**Figure 3:**
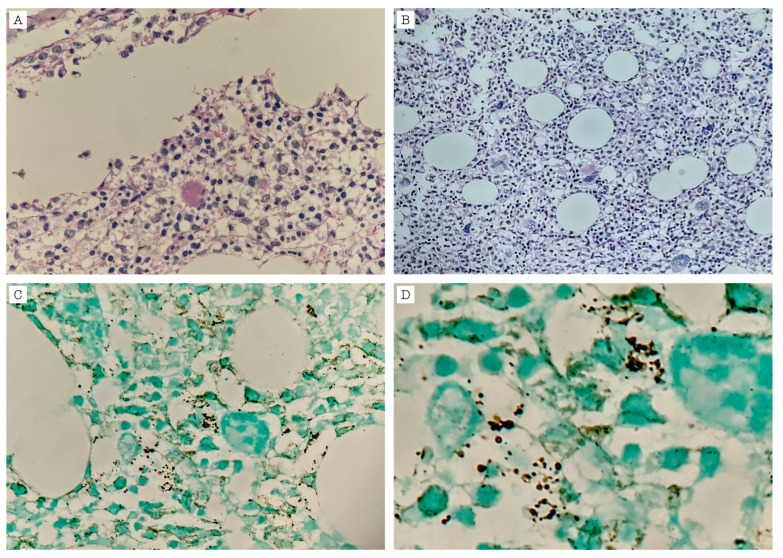
Bone marrow biopsy with, as follows: (**A**,**B**) Hematoxylin-eosin stain revealing foamy amorphic exudate; and (**C**,**D**) GMS (Grocott–Gömöri’s methenamine silver) stain and magnification revealing dark, round small structures with no budding within myeloid and erythroid cells suggestive of *P. jirovecii* yeasts.

During hospitalization, the patient developed pancytopenia, prompting a bone marrow biopsy, which revealed foamy, amorphous eosinophilic material and small, non-budding round structures suggestive of *P. jirovecii* yeasts (Figure 3), confirming disseminated infection with bone marrow involvement. A diagnosis of KS was confirmed by a histopathologic analysis of both a skin biopsy and an inguinal lymph node biopsy. Additionally, video capsule endoscopy revealed lesions consistent with KS involving the esophagus, duodenum, jejunum, and ileum. By that time, therapy with cotrimoxazole had been completed for a total of 21 days, with a progressive resolution of leukopenia and thrombocytopenia, and he was referred to an oncology institution to complete oncologic and antiretroviral treatment. Two months later, the patient was hospitalized again due to clinical deterioration, ultimately succumbing to multisystem organ failure.

## 3. Discussion

*P. jirovecii* infection presents in patients with immunosuppressive conditions such as AIDS, hematologic or solid organ malignancies, and iatrogenic immunosuppression [1,2]. This infection primarily affects the lungs, as the fungus adheres to and erodes type 1 pneumocytes, leading to diffuse alveolar damage [15]. In immunocompetent individuals, *P. jirovecii* colonization is typically asymptomatic and self-limited. This effective control is mediated through the coordinated activation of antigen-presenting cells, CD4+ T cells, and B cells within bronchus-associated lymphoid tissue. These structures facilitate communication between CD4+ T cells—particularly follicular helper T cells—and B cells, leading to M2 macrophage polarization and fungal clearance with minimal inflammation [16]. In contrast, immunocompromised hosts—especially those with CD4+ T cell depletion—fail to mount this organized response. The fungus proliferates in the alveoli, triggering a poorly regulated, pro-inflammatory reaction. In this context, CD8+ cytotoxic T cells, NK cells, and pro-inflammatory Th1 responses predominate, promoting M1 macrophage activation through IFN-γ and TNF-α. While these pathways enhance fungal clearance, they also contribute significantly to pulmonary inflammation and tissue damage. Furthermore, *P. jirovecii* surface antigens such as major surface glycoprotein (Msg) and β-D-glucan stimulate Toll-like and C-type lectin receptors, enhancing local inflammation and impairing surfactant production, thus compromising alveolar integrity and gas exchange [16,17].

The most common symptoms are dry cough, progressive dyspnea, fever, and low exercise tolerance [15,18]. The incubation period from infection to the onset of clinical disease is approximately 5 to 6 days but sometimes can be extended up to 28 days [15]. Upon consultation, most patients present with hypoxemia, a high alveolar–arterial gradient, and in some cases, respiratory failure [15,18].

Diagnosing PCP requires a susceptible immunosuppressed host with a compatible clinical syndrome, and suggestive laboratory and imaging findings [19]. Chest computed tomography is the imaging modality of choice and usually reveals diffuse ground glass lung opacities, sometimes accompanied by lobar consolidations and subpleural compromise. In contrast, other less frequent tomographic findings, such as cysts or pneumothorax, can also be found [19,20]. Laboratory diagnosis usually relies on indirect methods such as a measurement of serum lactate dehydrogenase [LDH] which is elevated in most cases of PCP in people living with HIV, due to its release from damaged lung tissue [19,21]. A sensitivity and specificity of 86% and 45.3% have been described, with a cut-off point of 268 UI/L [19,21]. Because *P. jirovecii* cannot be cultured ex-vivo, diagnosis depends on a direct visualization of the fungus or molecular detection of its DNA [19,21]. Currently, the gold standard method is the analysis of bronchoalveolar lavage, with a reported sensitivity of 95–100% [15,19,21]. A diagnosis can be made with the observation of the trophic or cystic forms with special stains such as Giemsa, Wright, Diff-Quik, Cresyl violet or Grocott–Gömöri’s methenamine silver; however, a diagnostic can also be made by the presence of eosinophilic amorphous exudates in the alveolar spaces, which can be observed with routine staining [15,19,21]. Specimens obtained through other techniques such as expectorated sputum, or induced sputum have a lower and variable performance [19,21]. The molecular diagnosis of PCP is based on detecting several gene loci with polymerase chain reaction [PCR] techniques, and performance improves by using multicopy targets such as Msg, mtLSU, HSP70, and cdc2 [22]. The sensitivity of these techniques can be improved by using nested PCR (nPCR). Since the fungus can colonize the airways without establishing infection, the specificity can be also improved with the use of quantitative PCR (qPCR), which can be performed on invasive and non-invasive specimens [22]. In people living with HIV, the detection of HSP70 by qPCR on bronchoalveolar lavage reported a sensitivity and specificity of 98% and 96%, respectively [23], and the detection of Msg by qPCR on oral wash reported a sensitivity and specificity of 88% and 85%, respectively [24]. A negative PCR will likely exclude PCP, especially in deep respiratory samples [25], but a positive PCR requires clinical interpretation to distinguish between colonization and active infection, and there is still a need for more research to validate and establish specific cut-off points for these molecular techniques [19,22,25].

Extrapulmonary infection by *P. jirovecii* is rare and the manifestations depend on the compromised organ [3]. The most commonly affected sites are the lymph nodes, liver, spleen, gastrointestinal tract, and bone marrow; with 64% of these cases reporting multiple organ involvement [3]. Excluding the bone marrow, tomographic studies often reveal hypodense lesions with a tendency to calcify on the affected organs, which progress to rimmed or complete calcifications after successful treatment [3]. The primary risk factor for developing an extrapulmonary infection is the AIDS stage of HIV infection, especially those patients who do not receive adequate prophylaxis with cotrimoxazole or receive other pharmacological regimens such as inhaled pentamidine [3,4,9,10,12]. Other risk factors include hypogammaglobulinemia and hematologic malignancies [3,23].

The mechanism by which *P. jirovecii* reaches the bone marrow remains unclear. Almost all patients with bone marrow compromise had a pulmonary infection first, which strongly suggests that the fungus reaches the bloodstream from the lungs [3,5,6,7,8,10,11,12]. It is believed that in severely immunosuppressed hosts, the microorganism reaches the pulmonary lymph nodes, escapes phagocytosis, and disseminates into the venous bloodstream through the efferent lymphatic drainage [5]. Alternatively, some histologic evidence suggests a possible mechanism through alveolar septal destruction and direct capillary wall invasion [5]. Upon reaching the bloodstream, the bone marrow compromise may represent just a stage of a disseminated infection, like what happens in miliary tuberculosis [5,26].

To our knowledge, bone marrow infection by *P. jirovecii* has been reported in only a small number of cases worldwide (Table 3) [3,4,5,6,7,8,9,10,11,12]. In our review, 63.6% of the affected patients were living with AIDS [3,5,6,8,9,10,11,12], while the remainder had various forms of lymphoma [4,7]. The mean age was 42 years, and 81.8% of the cases occurred in male patients. [3,4,5,6,7,8,9,10,11,12]. The most frequent symptoms were dyspnea (45.4%) and fever (36.3%). Blood cytopenias such as anemia, leukopenia, and thrombocytopenia were described in almost all of these cases [3,4,5,7,8,10,12], as well as primary pulmonary infection, which supports the hypothesis that *P. jirovecii* reaches the bloodstream from the lungs through the efferent lymphatic drainage after escaping phagocytosis, or by a direct invasion of capillaries [3,5,6,7,8,10,11,12]. All these cases were diagnosed with a histologic examination of the bone marrow, and the most frequent findings were the presence of amorphous eosinophilic exudates or *P. jirovecii* cysts [3,4,5,6,7,8,10,11,12]. Treatment regimens were variable, and a high mortality rate greater than 90% was described despite treatment [3,4,5,6,7,8,9,10,11,12].

In this case, while the diagnosis of disseminated *P. jirovecii* infection was confirmed histologically through bone marrow biopsy, the subsequent clinical deterioration and death raise the possibility of additional or alternative diagnoses. One potential contributor is KS-IRIS, particularly if antiretroviral therapy was initiated after discharge. KS-IRIS is known to present with a rapid progression of KS lesions and systemic inflammatory features, particularly in the presence of pulmonary involvement, which carries a poor prognosis. Another possible diagnosis is KICS, a hyperinflammatory state driven by HHV-8 replication that often overlaps clinically with multicentric Castleman disease [14]. KICS is characterized by features such as pancytopenia, SIRS, and hyponatremia, all of which were present in this case. Additionally, hemophagocytic lymphohistiocytosis (HLH) must be considered. HLH is a hyperinflammatory syndrome that can occur in the setting of HIV, opportunistic infections, or malignancies such as KS. The presence of persistent fever, pancytopenia, liver dysfunction, and inflammatory markers raises clinical suspicion [27]. However, we lacked key diagnostic parameters such as ferritin, triglycerides, and soluble IL-2 receptor levels, and hemophagocytosis was not documented in the bone marrow biopsy which lowers the probability. These conditions may account for the patient’s rapid clinical decline despite appropriate antimicrobial therapy and are supported by rising inflammatory markers such as leukocytosis and elevated C-reactive protein levels.

Unfortunately, due to the patient’s transfer to another institution, we did not have access to clinical records from the final hospitalization, and no autopsy was performed. Thus, we were unable to confirm whether ART had been initiated, or whether there were additional clinical, laboratory, or imaging findings that might have supported a diagnosis of KS-IRIS or KICS. These limitations preclude definitive conclusions regarding the cause of death and highlight the diagnostic complexity in severely immunocompromised patients with overlapping opportunistic infections and malignancies.

Our case shares several features with the few reported instances of *P. jirovecii* involvement of the bone marrow in patients with advanced AIDS. As in previous reports, the patient exhibited profound immunosuppression, with a markedly low CD4 count and a high HIV viral load—both recognized risk factors for extrapulmonary dissemination. Notably, bone marrow involvement in our case was confirmed antemortem via bone marrow biopsy, whereas most previously documented cases were identified postmortem, often incidentally. This distinction reinforces the notion that disseminated *P. jirovecii* infection involving the bone marrow is likely underdiagnosed during life, particularly in the absence of targeted histopathologic evaluation.

## 4. Conclusions

*P. jirovecii* infection typically presents as pulmonary disease but may occasionally disseminate to extrapulmonary sites such as the bone marrow, particularly in patients with advanced immunosuppression. Bone marrow involvement is an uncommon but clinically significant finding that is usually identified through histopathologic evaluation and is associated with a high mortality.

This case contributes to the limited literature on disseminated *P. jirovecii* infection and highlights the importance of considering bone marrow involvement in people living with HIV who present with PCP and unexplained cytopenias. To date, no clinical trials have defined optimal treatment strategies for extrapulmonary or disseminated *P. jirovecii* infection, and it remains uncertain whether the standard 21-day cotrimoxazole regimen for pulmonary disease is adequate in these scenarios. Ongoing clinical awareness, histopathologic confirmation when feasible, and individualized clinical judgment remain essential in managing these rare and challenging presentations.

## Figures and Tables

**Table 1:** Laboratory findings.

Laboratory	Reference Values	Admission	Readmission (5 Days After Discharge)	Second Discharge (38 Days After Readmission)
White blood cell count (×10^3^/μL)	4.8–10.00	5.1	11.65	7.3
Neutrophils (×10^3^/μL)	1.40–6.50	3.03	9.84	5.4
Lymphocytes (×10^3^/μL)	0.80–4.00	1.54	1.13	1.37
Monocytes (×10^3^/μL)	0.00–0.70	0.36	0.66	0.47
Eosinophils (×10^3^/μL)	0.00–2.00	0.15	0	0.03
Hematocrit (%)	45.0–54.0	48.5	50.2	33.5
Hemoglobin (g/dL)	14.0–18.0	16.5	17	11.2
Platelets (×10^3^/μL)	150–450	141	70	303
C-reactive Protein (mg/L)	0.00–10.00	22.4	76.06	
Lactate Dehydrogenase (U/L)	98.00–192.00	331		
Total Bilirubin (mg/dL)	0.30–1.20		15.3	2.54
Direct bilirubin (mg/dL)	0.10–0.50		10.2	1.17
Alanine aminotransferase (U/L)	17.00–63.00		75.5	31.7
Aspartate aminotransferase (U/L)	15.00–41.00		104.12	44.3
Blood urea nitrogen (mg/dL)	6.00–20.00	8.74	17.4	25.2
Creatinine (mg/dL)	0.61–1.24	0.39	0.92	0.85
pH	7.350–7.450	7.45	7.51	
SaO_2_ (%)	80.0–100.0	92.6	93	
PaO_2_ (mmHg)		75.2	66	
PCO_2_ (mmHg)	26.0–50.0	31	26	
HCO_3_ (mmol/L)	18.0–23.0	21.4	21	
FiO_2_ (%)		40	21	
PaO_2_/FiO_2_		188	314	
Alveolar–arterial gradient		91.25	9.23	

**Table 2:** A summary of infectious data.

Laboratory	Specimen	Results
Aerobic and anaerobic cultures	Blood	Negative
*Treponema pallidum* (VDRL)	Blood	Positive (1:4 dilutions)
HIV antibodies	Blood	Positive
Human immunodeficiency virus viral load (copies/mL)	Blood	Positive (2,095,776)
Lymphocytes CD4 (cells/mm^3^)	Blood	33
Lymphocytes CD45 (cells/mm^3^)	Blood	524
Lymphocytes CD3 (cells/mm^3^)	Blood	368
Lymphocytes CD8 (cells/mm^3^)	Blood	305
Hepatitis A antibodies	Blood	Negative
Hepatitis B antigen	Blood	Negative
Hepatitis C antibodies	Blood	Negative
Cytomegalovirus IgG antibodies	Blood	Positive (30.3)
Cytomegalovirus IgM antibodies	Blood	Negative
Typhoid O Antibodies (latex agglutination)	Blood	Negative
Typhoid H Antibodies (latex agglutination)	Blood	Negative
*Brucella abortus* Antibodies (latex agglutination)	Blood	Negative
*Aspergillus* Galactomannan Antigen (lateral flow assay)	Blood	Negative
*Mycobacterium tuberculosis* PCR (GeneXpert Ultra)	Sputum	Negative
*Leptospira* IgM Antibodies	Blood	Negative
*Histoplasma* Antigen	Urine	Negative
*Cryptococcus* Antigen (latex agglutination)	Blood	Negative
Epstein Barr IgG Antibodies	Blood	Positive
Epstein Barr IgM Antibodies	Blood	Negative
Ziehl-Neelsen	Bronchoalveolar lavage	Negative
*Mycobacterium tuberculosis* PCR (GeneXpert Ultra)	Bronchoalveolar lavage	Negative
Direct Fungal Microscopy	Bronchoalveolar lavage	Negative
*Aspergillus* Galactomannan Antigen	Bronchoalveolar lavage	Negative
Mycobacteria Growth Indicator Tube	Bronchoalveolar lavage	Negative
Fungal Culture	Bronchoalveolar lavage	Negative
Common Microorganisms Culture	Bronchoalveolar lavage	Negative

**Table 3:** Cases of *P. jirovecii* bone marrow infection reported worldwide.

Author and Year	Sex and Age	Clinical Manifestations	Hematological Manifestations	Medical Records	Extramedullary Infection	Concomitant Conditions	Diagnosis	Treatment	Outcomes
Rossi, J.F., et al. (1985) [7]	F/75	Cervical mass, signs and symptoms of hypercalcemia	Pancytopenia	Lymphoplasmacytic lymphoma in chemotherapy	PCP (postmortem findings)	*Candida albicans* septicemia	Bone marrow biopsy (G with amorphous eosinophilic material and some organisms with a central red nucleus and pale blue cytoplasm were seen, GMS with PJ cystic walls)	ND	Death
	M/36	Respiratory failure	Anemia, severe thrombocytopenia	Hodgkin’s disease in chemotherapy and radiotherapy, Hip osteonecrosis, Pulmonary fibrosis	PCP (first)	*Bacteroides melaninogenica* infection in hip	Bone marrow biopsy (G with amorphous eosinophilic material and some organisms with a central red nucleus and pale blue cytoplasm were seen, GMS with PJ cystic walls)	ND	Death
Heyman, M.R., et al. (1987) [5]	M/34	Weight loss, dyspnea, arthralgias, myalgias, malaise, anorexia, hair loss, fever (38.2 °C), night sweats and decreasing visual acuity	Pancytopenia	AIDS (CD4 count and viral load unknown), diabetes	PCP (first)	CMV retinitis, oral candidiasis, *Mycobacterium avium intracellulare* infection in bone marrow and lungs; CMV infection in lungs and liver (postmortem findings), *Mycobacterium* infection in liver, colon and lymph nodes (postmortem findings)	Bone marrow biopsy (GMS with numerous extracellular PJ organisms)	Cotrimoxazole	Death
Unger, P.D., et al. (1988) [8]	M/40	Decreasing visual acuity, progressive confusion and withdrawal, decreased urinary output, and severe dyspnea	Anemia	AIDS (CD4 count and viral load unknown), KS	PCP (first), PJ infection of spleen, lymph nodes, adrenal gland, Virchow–Robin spaces (postmortem findings)	*Staphylococcus aureus* sepsis, KS	Post-mortem histologic examination of bone marrow (GMS with PJ cysts in characteristic ovoid, crescentic, and helmet-shaped configurations, MC stain was negative)	ND	Death
Lubat, E., et al. (1990) [9]	ND	ND	ND	ND	ND	ND	Bone marrow biopsy	ND	ND
Amin, M.B., et al. (1990) [10]	M/29	Dyspnea, weakness, progressive cough, fever, cervical adenopathy	Pancytopenia	AIDS (CD4 count and viral load unknown)	PCP (first), PJ infection of lymph nodes, liver, spleen and kidneys (postmortem findings)	None	Post-mortem histologic examination of bone marrow (GMS with PJ cysts and amorphous exudates)	Dapsone, azidothymidine, aerosolized pentamidine, and aspirin (cotrimoxazole allergy)	Death
Telzak, E.E., et al. (1990) [3]	M/33	Fever, chest pain, lethargy	Pancytopenia	AIDS (CD4 count and viral load unknown), KS, *Candida* esophagitis, Herpes zoster of the neck and jaw, CMV retinitis	PCP (first), PJ infection in liver, spleen, adrenals, kidneys and pituitary (postmortem findings)	KS involving the skin, trachea, lungs, duodenum, lymph nodes, and bone marrow; *Mycobacterium avium intracellulare* infection in lungs, lymph nodes and spleen; CMV infection in adrenal medulla (postmortem findings)	Post-mortem histologic examination of bone marrow (H-E with fluffy eosinophilic material and GMS with complete replacement of hematopoietic elements by dark round structures suggestive of PJ cysts)	Broad-spectrum antibiotics	Death
Rossi, J.F., et al. (1990) [6]	M/74	Respiratory failure	ND	Alcoholic cirrhosis, in situ gastric neoplasm, AIDS (T CD4/CD8 0.7 ratio)	PCP (first)	ND	Bone marrow biopsy (G with foamy eosinophilic material, GMS with PJ cystic walls)	ND	Death
Momose, H., et al. (1991) [11]	M/35	Fatigue and dyspnea	ND	AIDS (CD4 count and viral load unknown), CMV retinitis, KS	PCP (first)	CMV retinitis, KS	Bone marrow biopsy (H-E stain with a focus of foamy eosinophilic exudate with otherwise unremarkable hematopoietic elements; GMS showed PJ cysts; Immunoperoxidase stain positive for PJ on the eosinophilic material)	Intravenous pentamidine switched to oral cotrimoxazole	Death
Grier, D.D., et al. (2009) [4]	F/34	Shortness of breath, abdominal distention, and lower extremity edema	Pancytopenia	Cutaneous T cell lymphoma	PJ infection of peritoneum and spleen	Disseminated *Candida* and CMV infections	Bone marrow biopsy (H-E stain with focal aggregates of foamy eosinophilic material; GMS stain demonstrated numerous cup-shaped organisms consistent with PJ)	ND	Death
Lerdlamyong, K., et al. (2017) [12]	M/45	Fever, weight loss	Anemia, leukopenia	AIDS (CD4 count 0.01 × 10^9^/L, viral load undetectable)	PCP (first)	Cryptococcal meningitis, CMV retinitis	Bone marrow biopsy (H-E stain, multiple small clusters of histiocytes containing several small yeast cells; GMS, numerous intracellular cup-shaped yeast cells without mucinous capsule, with lack of budding; MC staining was negative)	Clindamycin and primaquine for 3 weeks, then dapsone for secondary prophylaxis (cotrimoxazole allergy)	Symptoms and hematologic compromise improved on treatment.
this work	M/34	Fatigue, diaphoresis, weight loss, dry cough, diarrhea, dyspnea	Thrombocytopenia with progression to pancytopenia	AIDS (CD4 Count: 33 cells/mL^3^. Viral load: 2,095,776 copies/mL)	PCP (first)	Syphilis, Oral candidiasis, KS	Bone marrow biopsy (H-E stain with foamy amorphic exudates; GMS with dark round small structures with no budding within myeloid and erythroid cells suggestive of PJ yeasts)	Cotrimoxazole for 21 days	Clinical improvement, died two months later

Abbreviations: CMV, Cytomegalovirus; G, Giemsa; GMS, Gömöri’s methenamine silver; H-E, Hematoxylin and eosin; KS, Kaposi’s sarcoma; MC, Mucicarmine; ND, Not Described; PCP, *Pneumocystis jirovecii* pneumonia; PJ, *Pneumocystis jirovecii*.

## Data Availability

All relevant information has been presented in the case report. Any additional data may be made available on reasonable request from the corresponding author.

## Abbreviations

The following abbreviations are used in this manuscript:

AIDS	Acquired immunodeficiency syndrome
CMV	Cytomegalovirus
DNA	Deoxyribonucleic acid
GMS	Grocott–Gömöri’s methenamine silver
H-E	Hematoxylin and eosin
HHV-8	Human herpesvirus 8
HIV	Human immunodeficiency virus
HLH	Hemophagocytic lymphohistiocytosis
KS	Kaposi’s sarcoma
LDH	Lactate dehydrogenase
MCD	Multicentric Castleman disease
Msg	Major surface glycoprotein
nPCR	Nested Polymerase chain reaction
PAS	Periodic Acid Schiff
PCP	*Pneumocystis jirovecii* pneumonia
PCR	Polymerase chain reaction
*P. Jirovecii*	*Pneumocystis jirovecii*
SIRS	Systemic inflammatory response syndrome
qPCR	Quantitative Polymerase chain reaction
VDRL	Venereal Disease Research Laboratory
